# Anti-inflammatory effects of a methanol extract of *Dictamnus dasycarpus* Turcz. root bark on imiquimod-induced psoriasis

**DOI:** 10.1186/s12906-019-2767-2

**Published:** 2019-12-02

**Authors:** Minjee Choi, Jun Koo Yi, Si-Yong Kim, Jung Hyun Ryu, Jinhee Lee, Wookbong Kwon, Soyoung Jang, Dongjun Kim, MyoungOk Kim, Hyungwoo Kim, Sung Hyun Kim, Seong-Kyoon Choi, Zae Young Ryoo

**Affiliations:** 1 School of Life Science, BK21 Plus KNU Creative Bioresearch Group, Kyungpook National University, 80 Daehakro, Bukgu, Daegu, 41566 South Korea; 2 Core Protein Resources Center, DGIST, Daegu, Republic of Korea; 3 Gyeongsangbukdo Livestock Research institute, Yeongju, South Korea; 4 Pusan National University School of Korean Medicine, Busan, South Korea; 5 School of Animal Science Biotechnology, Kyungpook National University, Daegu, South Korea; 6 Department of Bio-Medical Analysis, Korea Polytechnic College, Chungnam, South Korea

**Keywords:** *Dictamnus dasycarpus*, Inflammation, Psoriasis, Skin, Gamma delta T cell, T helper cell

## Abstract

**Background:**

The root bark of *Dictamnus dasycarpus* Turcz. has been successfully used for the treatment of inflammatory skin conditions such as eczema and pruritus. However, the anti-psoriatic effect of this plant has not until now been investigated.

**Methods:**

The aim of this project was to investigate whether a methanol extract of *Dictamnus dasycarpus* Turcz. root bark (MEDD) can be used as a therapeutic agent for psoriasis in C57BL/6 mice model of imiquimod (IMQ)-induced psoriasis. IMQ and MEDD was applied to mouse skin continuously for 7 days. The skin phenotype and the levels of inflammatory cytokines, such as interferon (IFN)-γ and interleukin (IL)-17, were analyzed. The immune cell population was determined by flow cytometry, and STAT1 and 3 protein levels were measured.

**Results:**

An alleviation of scaly skin phenotype, immune cell infiltration in the dermis, and epidermal hyperplasia was observed after daily MEDD treatment in the lesion-affected area. It was also found that MEDD reduced IL-17 cytokine levels decreased by 44.37% (*p* < 0.05), the number of IL-17-producing Th17 cells and γδT cells, and the size of the Th1 population secreting IFN-γ decreased by 45.98, 62.21, and 44.42%, respectively (*p* < 0.05), compared with the vehicle control group. STAT3 signals, associated with IL-17 are also reduced by MEDD.

**Conclusions:**

An anti-psoriatic effect of MEDD was observed, as determined by decreased skin inflammation, reduced number of inflammatory cytokines, and a smaller population of inflammatory cells. These results contribute to the validation of the use of MEDD in the treatment of psoriasis.

## Background

Psoriasis is a chronic inflammatory skin disease characterized by epidermal keratinocyte hyper-proliferation caused by crosstalk between keratinocytes and immune cells [1], resulting in epidermal hyperplasia (acanthosis), retention of keratinocyte nuclei in the stratum corneum (parakeratosis), neutrophilic exudates in the epidermis (Munro’s microabscess), and elongated rete ridges [2]. Many pro-inflammatory cytokines and chemokines, including tumor necrosis factor (TNF)-α, interleukin (IL)-6, and IL-22, are associated with the aggravation of psoriatic lesions [3]. IL-17, which is produced mainly by T helper 17 (Th17) cells and γδT-cells, increases the proliferation of keratinocytes and augments keratinocyte gene expression involved in the immune response [4]. Increased levels of IL-17 in serum and at lesion sites have been reported in patients with psoriasis compared with those in healthy controls. Levels of this cytokine are correlated with disease severity [5]. T helper 1 (Th1) cells are the major producers of IFN-γ. IFN-γ has been shown to be a potent promoter of Th17 cell function and trafficking of Th17 cells to immune sites, thereby exacerbating the pathogenesis of psoriasis [6].

IL-17 and IFN-γ are known to be regulated by the signal transducer STAT3 [7] and activator of transcription STAT1 [8] . Dysregulated STAT signaling is associated with chronic inflammatory disease and autoimmunity, and therefore STAT is a potential therapeutic target for this condition.

*Dictamnus dasycarpus* Turcz., a member of the family *Rutaceae*, is a perennial herbal plant widely distributed throughout Eastern Asia [9, 10]. As a traditional remedy, the root bark of this plant has been used to treat skin diseases such as eczema, pruritus, urticaria, and atopic dermatitis [11]. The root bark of this plant contains many bio-active components, such as prenylated flavanone, limonoids, furoquinoline alkaloids, quinoline alkaloids, pyrrolidine alkaloids, sesquiterpenes, coumarins, and phenolic glycosides [12–19]. Previous studies have shown that extracts of this plant have anti-allergenic [20] and anti-inflammatory effects, including reduction of immune cell activity [21], proliferation [10], and recruitment [22]. However, the anti-inflammatory effects of this plant on psoriasis have not previously been studied.

The aim of this research was to investigate the effects of a methanol extract of *Dictamnus dasycarpus* Turcz., root bark (MEDD) in a mouse model of imiquimod (IMQ)-induced psoriasis. Therefore, the number of inflammatory cytokine immune cells was measured and the phenotype of the skin observed.

## Materials and methods

### Chemicals and reagents

Ethanol (Merck, Germany), acetone (Duksan, Korea), hair removal cream (Veet; Reckitt Benckiser, France), IMQ (Dong-a Otsuka, Korea), Vaseline (Unilever Korea, Korea), 70 μm strainers (SPL life science, Korea), IL-17 ELISA kits (R&D Systems MN, USA), and PerCP-Cy5.5-Rat IgG1,κ (RTK2071, Biolegend, USA) were used. First Strand cDNA Synthesis Kits and SYBR Premix EX Taq were purchased from Takara Bio (Japan).

CD16/CD32, fluorescein isothiocyanate (FITC)-anti-mouse CD4 (Mouse CTL clone V4) and FITC-Rat IgG2b,κ (TNP-Keyhole Limpet Hemocyanin) were purchased from BD Biosciences (USA). FITC-anti-mouse γδ TCR (UC7-13D5) and phycoerythrin (PE)-anti-mouse IL-17A, FITC-Armenian Hamster IgG (eBio299Arm), PE-anti-mouse IL-17A (eBio17B7), PerCP-Cy5.5-anti-mouse IFNγ (XMG1.2), PE-anti-Rat IgG2a/κ (eBR2a) and TRIzol reagent were purchased from Thermo Fisher Scientific (USA). Rabbit anti-STAT1(D1K9Y), rabbit anti-phospho-STAT1 (Tyr701), rabbit anti-STAT3 (79D7), rabbit anti-phospho-STAT3 (D3A6), rabbit anti-STAT5 (D2O6Y), rabbit anti-phospho-STAT5 (C11C5) and HRP-conjugated anti-rabbit IgG antibodies were purchased from Cell Signaling (USA). Mouse anti-β-Actin (C4) antibodies and HRP-conjugated m-IgGκ BP were purchased from Santa Cruz Biotechnology (USA).

### Animals

Because IMQ is known to cause more severe inflammation in female mice than in male mice [23] used seven- to nine-week-old female wild-type C57BL/6 mice were used (Hyochang, Daegu, Korea). All animal experiments were performed according to the guidelines for animal experimentation and with permission from the Animal Use and Care Committee of Kyungpook National University (approval No.2018–0038). The mice were raised and maintained under conventional conditions in a room with a 12-h light/dark cycle, at a temperature of 23 °C, and were given free access to food and water. A minimum number of mice was used, to comply with the principles of replacement, refinement and reduction (the 3Rs).

### MEDD preparation

The root bark of Dictamnus dasycarpus Turcz. was purchased from Kwang Myung Dang Medicinal Herbs (Ulsan, Korea). Before extraction, plant specimen was authenticated by professor Hyungwoo Kim, one of the authors of this article. MEDD (Voucher no.MH2010–010) was kindly provided by professor Hyungwoo Kim [11]. MEDD was deposited at the Division of Pharmacology, School of Korean Medicine, Pusan National University.

Before starting the experiment, 15 mg MEDD was dissolved in 1 mL ethanol. The 1 ml of ethanol with dissolved MEDD, and 1 ml of ethanol alone as a vehicle control, were diluted with 4 ml of acetone-olive oil (4:1) solution.

### Experimental design of IMQ-induced psoriasis and MEDD treatment

Mice were anesthetized with Avertin (2.5% tribromoethanol, intraperitoneally, 100 μL/10 g body weight). The dorsal skin of anesthetized mice was depilated using surgical scissors and hair removal cream. After depilation, mice were placed in individual cages. Mice were used in the experiment after resting for a day. The depilated mouse dorsal skin was treated daily for seven consecutive days with 62.5 mg Aldara cream containing 5% IMQ [24]. As a control for IMQ, the same volume of Vaseline was applied to the skin. After 6 h of IMQ treatment, 180 μg/60 *μ*L of MEDD or the same volume of vehicle control was applied daily to the depilated dorsal skin. The concentration of MEDD was treated at the same concentration that had previously had an anti-allergic effect on mice skin in the previous experiment [11]. One day after the last treatment, the anesthetized mice were photographed for identification of the skin phenotype, and then euthanized by carbon dioxide inhalation. The home cage was filled with carbon dioxide through a regulator connected to a lid that fit on the top of the home cage, at a flow rate displacing 20% of the cage air per minute. The dorsal skin, draining lymph nodes and serum were collected from euthanized mice tissues for subsequent analysis. The body and spleen were weighed, as a measure of the severity of inflammation. A total of 20 mice were used. The mice used were divided into 4 groups and used as follows: 4 Vaseline, 5 IMQ, 4 IMQ + Vh(vehicle), 7 IMQ + MEDD. All experiments were performed in the laboratory.

### Histological analysis

Mouse dorsal skin was fixed with 4% paraformaldehyde and embedded in paraffin. Tissue sections with a thickness of 5 μm were stained with hematoxylin and eosin (H&E). The thickness of the epidermis was measured based on the thin area between rete ridges, sebaceous glands, and hair follicles using Leica Application Suite software. The epidermis was measured four times per slide, for four slides per mouse. Using a grading scale of 0 to 4, the severity of psoriasis was determined separately for three different areas: epidermal thickness, immune cell infiltration, and parakeratosis. Disease assessments were performed in a blinded manner by four researchers.

### Flow cytometry

After isolation of draining lymph nodes, each was passed through a 70 *μ*m strainer to obtain single cells. Red blood cells were removed, and the cells were fixed with 4% paraformaldehyde for 10 min. Permeabilization was carried out on ice for 30 min using 90% methanol. To block Fc receptors, cells were reacted with anti CD16/CD32 for 5 min before staining. FITC-anti-mouse CD4, PE-anti-mouse IL-17A, PerCP-Cy5.5-anti-mouse IFNγ, FITC-Rat IgG2b,κ, PE-anti-Rat IgG2a/κ, and PerCP-Cy5.5-Rat IgG1,κ antibodies were used to identify the populations of Th1 and Th17 cells. FITC-anti-mouse γδ TCR and PE-anti-mouse IL-17A, FITC-Armenian Hamster IgG were used to analyze the population of γδ T cells producing IL-17. The cells were analyzed using a FACSAria III flow cytometer (BD Biosciences).

### Quantitative real time PCR

Total RNA was extracted from skin lysates using TRIzol reagent, according to the manufacturer’s protocol. The cDNA from total RNAs was synthesized using First Strand cDNA Synthesis Kits and measured using quantitative PCR with SYBR Premix EX Taq. To investigate the levels of mRNA for inflammatory cytokines (*Tnf-α, Ifng, Il6, Il17a, Il22,* and *Ccl2*), real-time PCR was performed using a Step One Plus PCR system (Applied Biosystems, CA, USA). The expression levels of the genes were normalized to the expression of the *Gapdh* gene in each sample.

### Elisa

IL-17 concentration in serum was measured using mouse IL-17 ELISA kit, according to the manufacturer’s protocols.

### Western blot analysis

The protein levels in the skin lysates were measured. Rabbit anti-STAT1, rabbit anti-phospho-STAT1, rabbit anti-STAT3, rabbit anti-phospho-STAT3, rabbit anti-STAT5, rabbit anti-phospho-STAT5 followed by HRP-conjugated anti-rabbit IgG antibodies were used. Mouse anti-β-Actin antibodies, followed by HRP-conjugated anti-mouse IgGκ BP antibodies were also used. Immunoblots were visualized using the ECL Detection System (GE Healthcare, Chicago, Illinois).

### Statistical analysis

The results are expressed as median ± interquartile range from at least three independent experiments. The significance of the differences between groups was calculated using Student’s *t*-test. In all analyses, a *p* value of less than 0.05 was considered to be statistically significant.

## Results

### Effects of MEDD on symptom severity in IMQ-induced psoriasis model mice

**Fig. 1:**
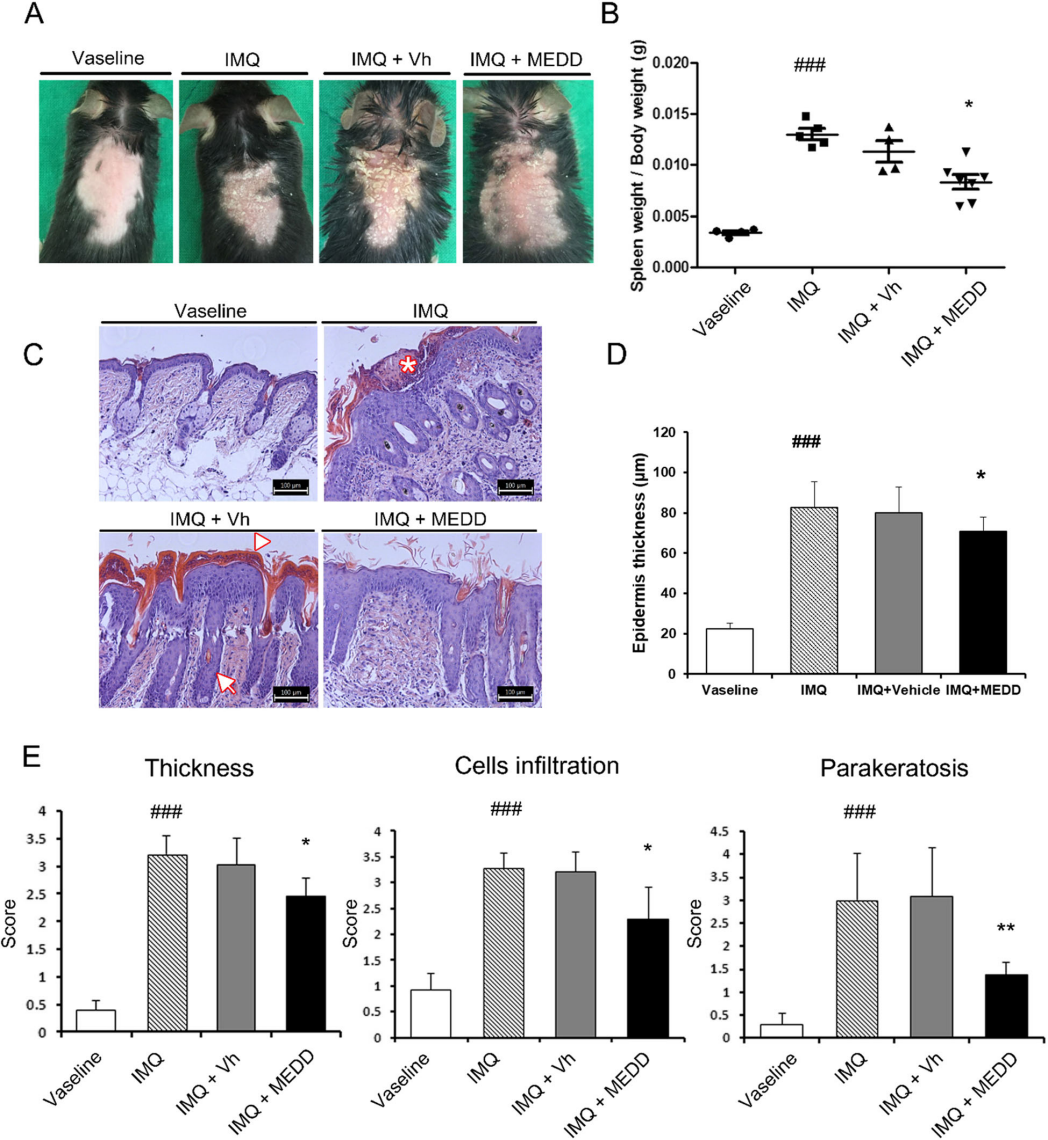
Effects of MEDD on symptom severity in IMQ-induced psoriasis model mice. The dorsal skin of mice was treated with Vaseline, imiquimod (IMQ), IMQ and Vh or MEDD for 7 days, and the phenotype was observed the next day. **a** Representative macroscopic views of the dorsal skin of each group of mice. **b** Calculation of spleen weight per body weight for each treatment group. Each dot represents one mouse. **c** Hematoxylin and eosin staining of paraffin sections from the dorsal skin of each group of mice. Note: Munro’s abscess (asterisk), parakeratosis (arrowhead), and rete ridge (arrow). Scale bar: 100 μm **d** Microscopic measurement of epidermal thickness (*μ*m) of dorsal skin. **e** Individual histological scoring of thickness, immune cell infiltration, and parakeratosis in the skin. **b**, **d**, **e** These results represent mean ± S.D. of four independent experiments. **a**, **c** The photograph is representative of four independent experiments. Statistical analysis was performed using Student’s *t*-test. * *p* < 0.05; ** *p* < 0.01, compared with mice in the IMQ- and vehicle-treated group; ###, *p* < 0.001, compared with Vaseline control mice; IMQ, imiquimod; Vh, Vehicle; MEDD, methanol extract of *Dictamnus dasycarpus* root bark.

To investigate the effect of MEDD on psoriasis, IMQ followed by MEDD or a vehicle control were applied to the dorsal skin. After 7 days of application, erythema and silver-whitish scales were noted in the IMQ- and vehicle-treated groups; the symptoms in the MEDD-treated group were alleviated (Fig. 1a).

Enlargement of the spleen is an index of the severity of inflammation [25], so the ratio of spleen weight to body weight was calculated for each group. The spleens of the IMQ-treated groups were significantly enlarged compared with those of the Vaseline controls. However, MEDD treatment significantly decreased spleen weight compared to the vehicle controls (Fig. 1b). To observe the symptoms histologically, H&E staining of the dorsal skin was performed 1 day after the last treatment. The IMQ and vehicle-treated groups showed thickened epidermis with elongated rete ridges (Fig. 1c arrow), parakeratosis (Fig. 1c arrowhead), and Munro’s abscess (Fig. 1c asterisk). These symptoms were alleviated in the MEDD-treated group (Fig. 1c).

The thickness of the epidermis was measured, as an indicator of the severity of the psoriasis. Epidermal thickness significantly increased after IMQ treatment compared with Vaseline controls, whereas MEDD significantly decreased epidermal thickness relative to vehicle controls (Fig. 1d).

Histopathological severity was quantified via scoring of epidermal thickness, immune cell infiltration in the dermis area, and degree of parakeratosis. The increased severity of inflammation in the IMQ and vehicle control groups was significantly decreased by the application of MEDD (Fig. 1e). MEDD did appear to alleviate IMQ-induced psoriasis-like symptoms.

### Effects of MEDD on inflammatory cytokine expression in IMQ-induced psoriasis

**Fig. 2:**
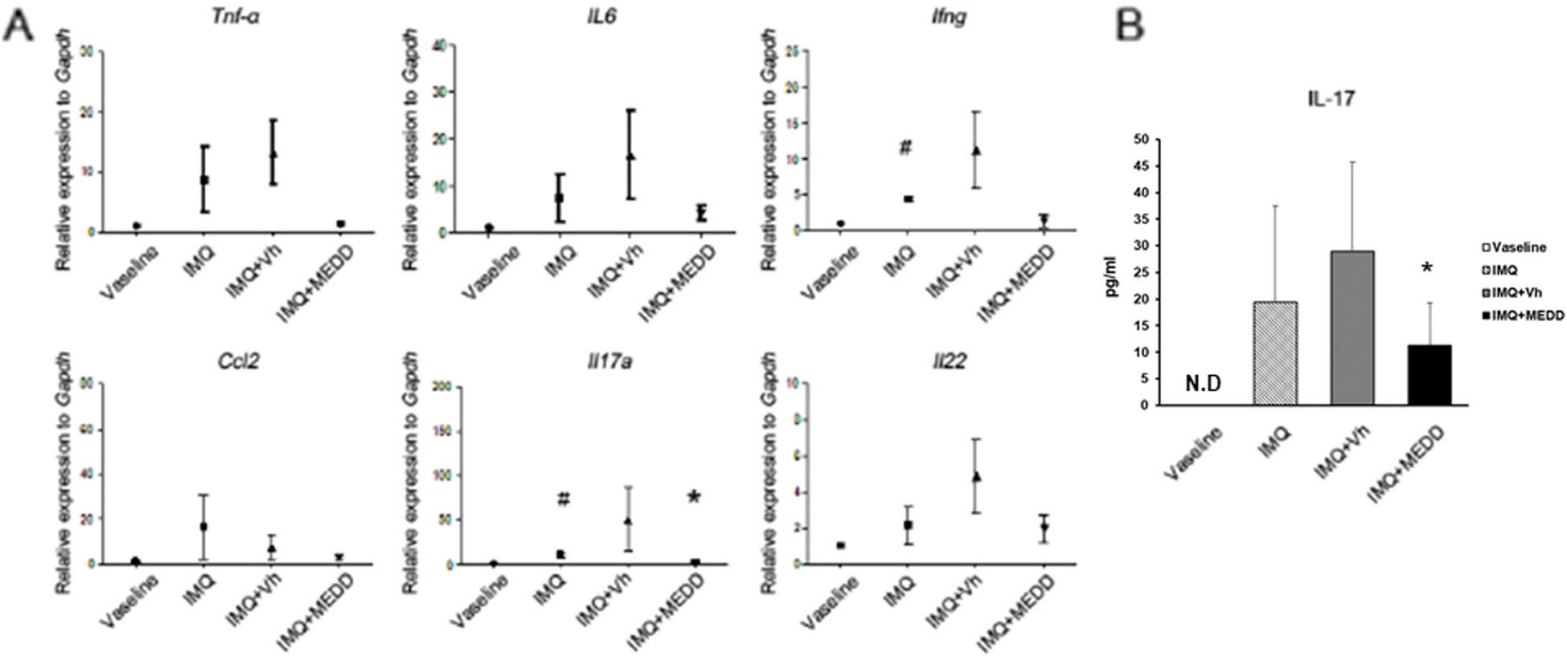
Effects of MEDD on inflammatory cytokine expression in IMQ-induced psoriasis. The dorsal skin of mice was treated with Vaseline, IMQ, IMQ and Vh or MEDD for 7 days, and cytokine levels were analyzed the next day. **a** mRNA levels of pro-inflammatory cytokines, *Tnf-α, Ifng, Il6, Il17a, Il22,* and *Ccl2* in dorsal skin. **b** IL-17A measurement in each group of mouse serum by ELISA. These results represent the mean ± S.D. of four independent experiments. Statistical analysis was performed using Student’s *t*-test. * *p* < 0.05, compared with mice in the IMQ- and vehicle-treated group; ### *p* < 0.001, compared with Vaseline control mice; IMQ, imiquimod; Vh, Vehicle; MEDD, methanol extract of *Dictamnus dasycarpus* root bark; N.D, Not detected.

To investigate the level of pro-inflammatory cytokines and chemokines in psoriatic dorsal skin lesions, real-time PCR was performed. IMQ treatment increased the level of mRNA for pro-inflammatory cytokines and chemokines, *Tnf-α, Il6, Il22,* and *Ccl2*, and significantly increased *Ifng* and *Il17a* compared to Vaseline controls. MEDD treatment decreased these pro-inflammatory cytokines, especially *Il17a,* compared with the vehicle controls (*p* < 0.05, Fig. 2a).

IL-17 levels in the serum were found to be increased by IMQ compared with the Vaseline controls. MEDD treatment significantly decreased cytokine expression compared with vehicle controls (Fig. 2b). These results indicate that MEDD reduced pro-inflammatory cytokines, especially IL-17 level in the skin and also in the whole body.

### Effect of MEDD on immune cell population in IMQ-induced psoriasis

**Fig. 3:**
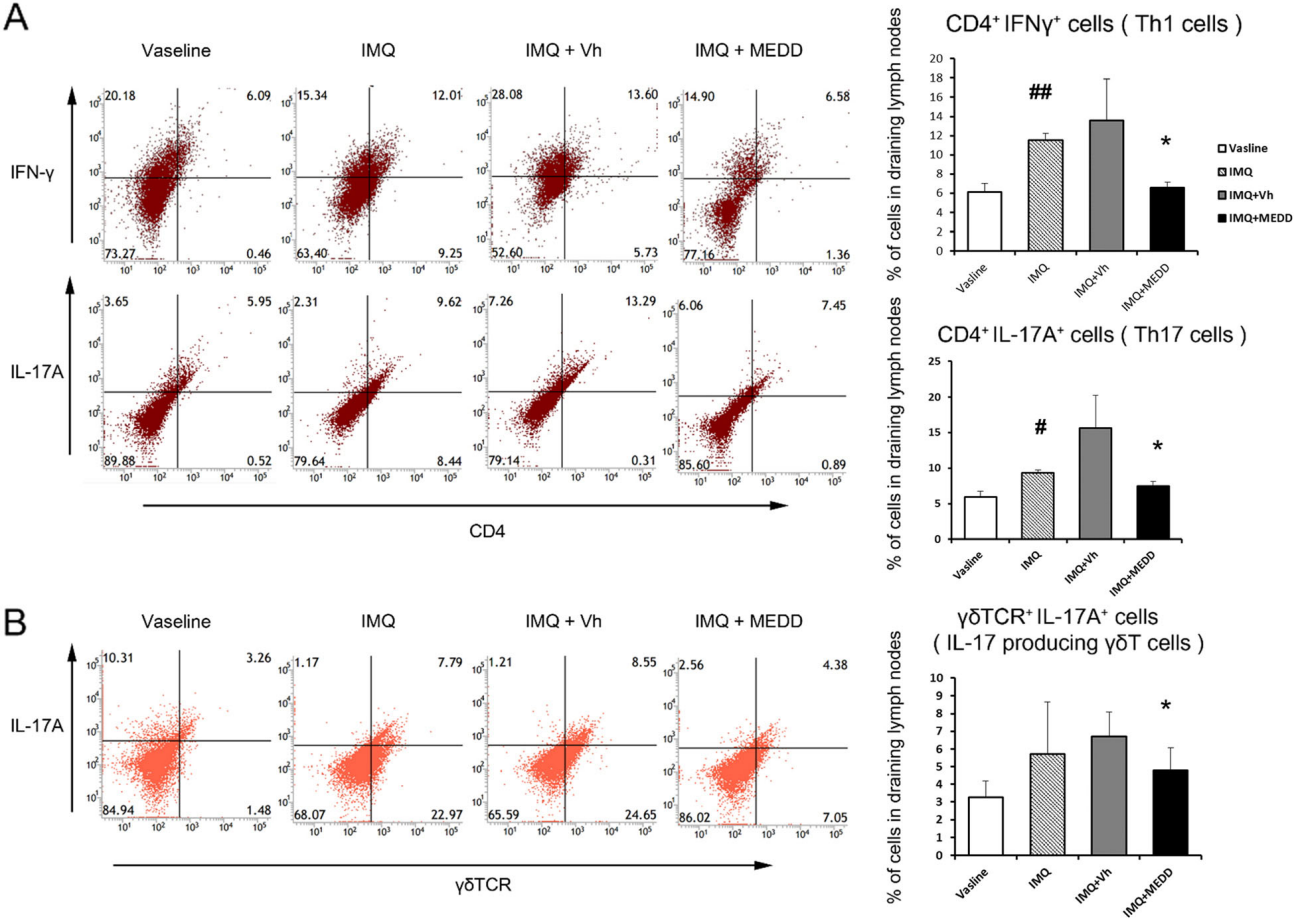
Effect of MEDD on immune cell population in IMQ-induced psoriasis The dorsal skin of mice was treated with Vaseline, IMQ, IMQ and Vh or MEDD for 7 days, and the immune cell population was analyzed by flow cytometry in the skin draining lymph nodes the next day. **a** CD4^+^IFNγ^+^ Th1 and CD4^+^ IL-17A^+^ Th17 cell populations were analyzed. (left) Representative dot blot and (right) quantified percentage of Th1 and Th17 cells. **b** IL-17A^+^ γδTCR^+^ (IL-17 producing γδT cells) cell populations were analyzed. (left) Representative dot blot and (right) quantified percentage of IL-17-producing γδ T cells. These results represent the mean ± S.D. of four independent experiments. Statistical analysis was performed using Student’s *t*-test. * *p* < 0.05, compared with mice in the IMQ- and vehicle-treated group; #, *p* < 0.05; ##, *p* < 0.01 compared with Vaseline control mice; IMQ, imiquimod; Vh, Vehicle; MEDD, methanol extract of *Dictamnus dasycarpus* root bark

Flow cytometry was used to investigate whether MEDD affects the populations of immune cells, the populations of CD4^+^IFNγ^+^ T helper 1 (Th1) cells and CD4^+^IL-17A^+^ T helper 17 (Th17) cells were increased by IMQ compared to the Vaseline controls and were significantly reduced by MEDD compared to the vehicle controls (Fig. 3a).

IL-17-producing γδT-cells are also associated with psoriasis [1]. Therefore, the population of IL-17^+^ γδTCR^+^ cells was measured. As in the Th17 cell population, the γδT cell population was significantly decreased by MEDD compared to the vehicle controls (Fig. 3b).

It can therefore be concluded that MEDD reduced the population of Th1, Th17, and IL-17 producing γδT-cells.

### MEDD effects on STAT1 and STAT3 in IMQ-induced psoriasis

**Fig. 4:**
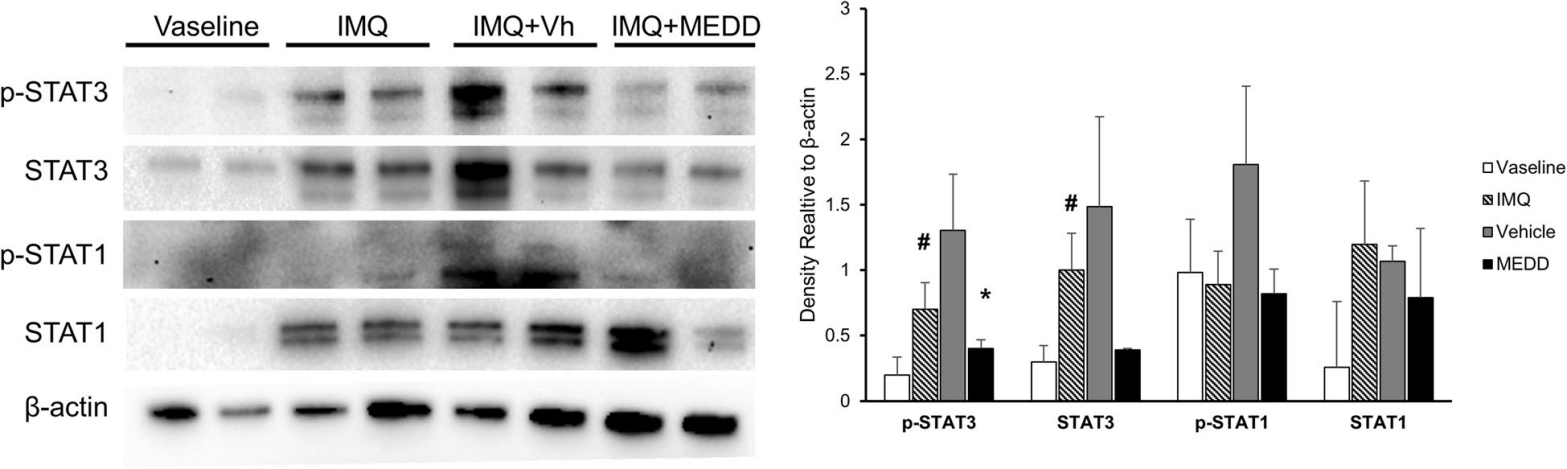
MEDD effects on STAT1 and STAT3 in IMQ-induced psoriasis The dorsal skin of mice was treated with Vaseline, IMQ, IMQ and Vh or MEDD for 7 days, and the immune cell population was analyzed by Western blotting in the skin lysates the next day. (left) Antibodies specific for pSTAT1, STAT1, pSTAT3, STAT3, and β-actin were used. Western blot performed on four samples per group. Among them, two samples per group were selected and are presented as representative images. (right) Densitometry quantification of proteins. Arrow indicates 86 (upper) and 79 (bottom) kDa size. These results represent mean ± S.D. of four independent experiments. Statistical analysis was performed using Student’s *t*-test. * *p* < 0.05, compared with mice in the IMQ- and vehicle-treated groups; #, *p* < 0.05 compared with Vaseline control mice; IMQ, imiquimod; Vh, Vehicle; MEDD, methanol extract of *Dictamnus dasycarpus* root bark.

STAT1 and STAT3 are the major factors that stimulate helper T cells to produce IFNγ and IL-17, respectively [26]. The levels of these STAT proteins were investigated using Western blot analysis of skin lysates. The amount of phosphorylated STAT3 and total levels of STAT3 increased in the IMQ-treated groups compared with Vaseline controls. After MEDD treatment, the levels of phosphorylated STAT3 and total amounts of STAT3 were decreased compared with vehicle controls. However, there was no significant decrease in the amount of STAT1 by MEDD treatment (Fig. 4). Therefore, it can be concluded that MEDD reduced the phosphorylation and total amount of STAT3.

## Discussion

This study produced evidence to indicate that there are anti-psoriasis effects of MEDD. Histological examination revealed that MEDD reduced the extent of scaly skin, decreased epidermal thickness, and alleviated parakeratosis. These symptoms are due to the hyper-proliferation of keratinocytes in psoriasis [3]. Therefore, it can be concluded that MEDD alleviated the hyperplasia of keratinocytes.

It was also evident that MEDD decreased the level of pro-inflammatory cytokines and the population of immune cells. Among the reduced inflammatory cytokines IL-17 was significantly reduced by MEDD compared with the vehicle controls. It was also apparent that MEDD reduced the numbers of IL-17-producing γδT cells, Th17 cells, and IFN-γ-producing Th1 cells. Previous studies have shown that MEDD reduces IFN-γ levels in allergic contact dermatitis [11]. However, this is the first study to show that MEDD downregulates IL-17 and decreases the population of immune cells producing IL-17.

MEDD reduced the total levels and phosphorylation of STAT3. Increased phosphorylation of STAT3 is known to stimulate the production of IL-17 [7]. Phosphorylation levels, as well as total amount, of these proteins is known to be associated with worsening of autoimmune diseases [27]. Therefore, decreased total protein levels of STAT3 is indicative of an anti-psoriasis effect of MEDD. Thus, it was demonstrated that MEDD inhibits IL-17 by decreasing STAT3. IL-17 is the major cause of deterioration in psoriasis. Therefore, it was concluded that MEDD relieved psoriasis symptoms by reducing the production of this cytokine.

The vehicle group had a more severe phenotype and greater inflammation than the IMQ-treated group. It is possible that the stress caused by the approximately 10 min of restraint needed to apply MEDD and vehicle to the lesion area on the mice increased inflammation [28]. In addition, repeated exposure of skin to acetone, a solvent in MEDD, may increase inflammation [29]. Despite these effects, the anti-inflammatory effect of MEDD on IMQ-induced psoriasis is apparent, because inflammation and phenotype are alleviated in the MEDD group compared to the vehicle group.

These anti-psoriatic effects of MEDD might be attributable to its bio-active components [12–19]. A previous study revealed that limonoids, quinoline alkaloids, and phenolic glycosides alleviate psoriasis symptoms [30–32]. The anti-psoriatic effects of MEDD may be caused by these bio-active components. The root bark of *Dictamnus dasycarpus* Turcz. has already been used extensively in East Asia [11]. Therefore, the anti-psoriasis effects observed in mice is expected to be transferrable to humans.

Also significant differences were obtained in this experiment even though a minimum number of mice were used to comply with the Replacement, Purification and Reduction Principle (3R).

Based on this study, it appears that MEDD has potential for use in the treatment of psoriasis.

## Conclusions

In this study, MEDD reduced the numbers of inflammatory cells and IL-17 by decreasing STAT1 and STAT3 in IMQ-induced psoriasis. These results demonstrate the anti-psoriasis effects of MEDD. This study indicates that MEDD may be valuable as a remedy for psoriasis.

## Data Availability

Not applicable. The datasets used and/or analyzed during the current study available from the corresponding author on reasonable request.

## Abbreviations

FITC: fluorescein isothiocyanate
IFN-γ: interferon-γ
IL-17: interleukin IL-17
IMQ: imiquimod
MEDD: methanol extract of *Dictamnus dasycarpus* Turcz. root bark
PE: phycoerythrin
STAT: signal transducer and activator of transcription
Th1: T helper 1
Th17: T helper 17
TNF-α: tumor necrosis factor (TNF)-α
